# Seeing the unseen: High-resolution AFM imaging captures antibiotic action in bacterial membranes

**DOI:** 10.1038/s41467-022-33839-z

**Published:** 2022-10-21

**Authors:** Telmo O. Paiva, Albertus Viljoen, Yves F. Dufrêne

**Affiliations:** Louvain Institute of Biomolecular Science and Technology, UCLouvain, Croix du Sud, Louvain-la-Neuve, Belgium

**Keywords:** Atomic force microscopy, Antimicrobials, Biochemistry

## Abstract

Advances in atomic force microscopy (AFM) techniques and methodologies for microbiology contribute to our understanding of the microbial cell surface. Recent studies show that AFM imaging of cells and membranes at (near) molecular resolution allows detailed visualization of membranes interacting with drugs.

## Atomic force microscopy as a tool for high-resolution bio-imaging

**Fig. 1:**
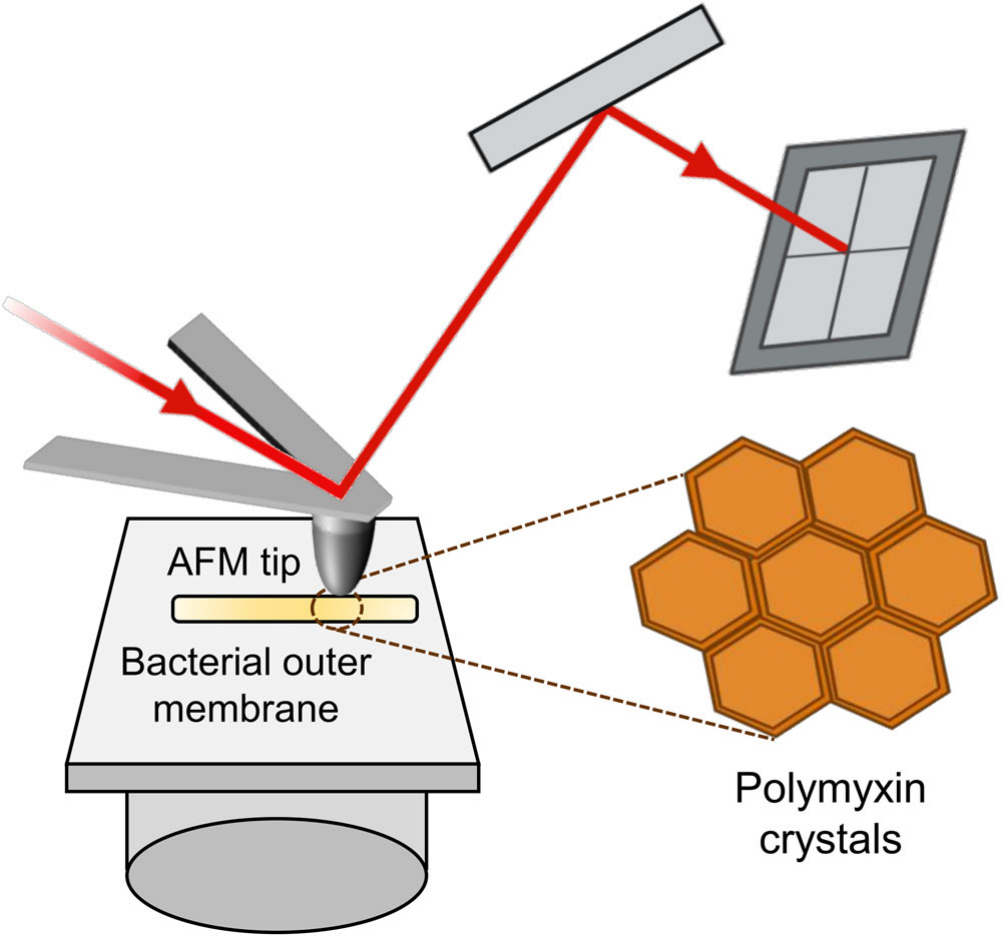
In AFM imaging a sample is scanned with a sharp tip attached to a soft cantilever whose up- and down-deformations are detected by a laser-photodiode system. Such AFM imaging has resolved the nanoscale crystal lattice formed by polymyxin in the bacterial outer membrane.

Owing to its capability of high-resolution imaging under physiological conditions, atomic force microscopy (AFM) has become an essential tool in investigating the structure and function of cell membranes, using both living cells and model biomembranes^1,2^. In AFM, a specimen is mounted on a piezoelectric scanner and scanned by a sharp tip attached to the end of a flexible cantilever (Fig. 1). Tip-sample interactions cause cantilever deflection, which is monitored by a laser beam that is focused on the free extremity of the cantilever and detected by a photodiode. AFM topographic imaging can be performed either in contact or in dynamic modes, depending on the way the tip interacts with the sample. While in contact mode the tip remains in contact with the sample; in dynamic mode it oscillates in the proximity of the sample, minimizing surface damage.

Over the years, AFM has been instrumental in unraveling the nanoscale architecture of bacterial membranes, thereby elegantly complementing optical nanoscopy techniques^3^. Bacteria are surrounded by cell envelopes consisting of an inner membrane, a cell wall made of peptidoglycan layers, and for Gram-negative bacteria, an outer membrane (OM). The cell envelope plays a central role in bacterial physiology, defining cell shape and division, helping bacteria resist turgor pressure, and exposing receptor sites for viruses and for cell adhesion. Moreover, cell envelope components are targets of some of the most efficient antibiotics.

## Atomic force microscopy reveals dynamic nature of microbial membranes

While AFM is able to reveal nanoscale structures directly on living cells, such as rodlet layers on fungal spores^4^ and the net-like structure formed by porin trimers on bacterial cells^5^, high resolution is generally achieved on isolated membranes or two-dimensional membrane protein crystals reconstituted in the presence of lipids^6^. Currently, a key challenge is to use advanced high-speed AFM to capture structural changes of bacterial membranes at high temporal resolution. In early work, the structure of individual bacteriorhodopsin trimers was identified at a speed of 100 ms per image^7^, and dynamic motions of monomers and trimers were visualized. Structural changes of bacteriorhodopsin were tracked in response to light within 1 s^8^ and insights for trimer-trimer interactions revealed^9^. The motion of about 70 OmpF trimers was tracked and individual subunits were resolved, revealing a wide distribution in the membrane due to diffusion-limited aggregation^10^. Interestingly, one can also use functionalized AFM tips to detect and localize specific proteins. For instance, Mulvihill et al.^11^ used malto-oligosaccharide-functionalized tips to image reconstituted trimers of the LamB maltoporin and simultaneously measure binding forces and frequencies of sugar ligands in single LamB pores. High-resolution topographs of this outer membrane protein allowed distinguishing LamB trimers exposing their extracellular side from ones exposing their periplasmic side. Sugar binding to LamB was purely asymmetric, taking place preferentially on the periplasmic side, contrary to what had been previously believed.

A particular strength of AFM is its ability to image living bacteria in real time while they grow or interact with antimicrobials. Either by characterizing mycobacteria growth and division in great detail^12^, or studying the kinetics of antimicrobial peptides on individual *E. coli* cells^13^, the Fantner group has been a pioneer in using high-resolution AFM to dynamically image live cells. Alsteens et al.^14^ studied the effect of antitubercular drugs on mycobateria, revealing major surface ultrastructural alterations. More recently, AFM was used to investigate the effects of the Mycobacterial membrane protein Large 3 (MmpL3) inhibitor, BM212, whose capacity to interfere with the highly hydrophobic nature of the mycobacterial cell wall was demonstrated by combining fast imaging with hydrophobic tips^15^.

Obtaining high resolution, molecular scale AFM images of bacterial surfaces interacting with drugs requires the use of isolated or reconstituted membranes. Due to their planar nature, supported lipid bilayers (SLBs) are particularly well suited for that purpose, as illustrated by Montero et al.^16^ who showed the formation of pores by ciprofloxacin and two derivatives in *E. coli* membranes. Recently, Parsons et al.^17^ used SLBs of *E. coli* lipid extracts to track the kinetics of pore formation by the membrane attack complex, a complement protein assembly.

## Atomic force microscopy to study antibiotic action

The widely studied *E. coli* outer membrane is composed of an inner leaflet of phospholipids and an outer leaflet rich in lipopolysaccharide (LPS) molecules, which, together with proteins, are the first targets encountered by antimicrobial drugs. The Hinterdorfer team^18^ studied the mode of action of polymyxin, an antibiotic targeting LPS. AFM images of LPS monolayers revealed an increase in roughness after antibiotic treatment, while functionalized tips were used to probe the strength of the polymyxin-LPS interaction. Manioglu et al.^19^, now report a detailed study in which the mechanism of action of this important drug is disclosed at the molecular level (Fig. 1). The authors elegantly combine high-resolution AFM imaging with structural biology and biochemical assays to demonstrate that polymyxin arranges LPS into crystalline structures to solidify the bacterial OM. By using *E. coli* membrane patches, compelling evidence is provided that polymyxins form hexagonal crystals along with LPS and divalent cations, which alter membrane thickness, area and stiffness, where the former property decreasing while the latter two increase. These changes weaken the bacterial OM, ultimately leading to its disruption. Crystal formation was evaluated for different polymyxin variants, including chemical modifications at the level of the ring and the tail of the molecule, and corresponding structure-activity relationship and minimal inhibitory concentration determined for each one of them.

While the biophysical changes of the OM reported by the authors clearly match the phenotypes previously identified for the polymyxin action, the identification of crystalline structures provides a mechanistic explanation for molecular dynamics simulation data published before. These findings will change the paradigm that polymyxins interact non-specifically with the bacterial membrane, but rather they *“have been shaped by evolution to form a specific high-order structure together with LPS”*. This study represents an important step forward in our understanding of how these drugs interact with LPS in Gram-negative bacteria, which will be essential to expand drug design to new variants of this antibiotic family.

Among the exciting challenges ahead, high-speed AFM should clearly help researchers to understand the mode of action of antibiotics, at unprecedented spatiotemporal resolution. In one such example, Zuttion et al.^20^ studied the activity of the commercial antimicrobial lipopeptide daptomycin on Gram-positive bacteria. This work combines both living and fixed bacteria, as well as both non-supported and supported model membranes, to study drug-membrane interactions. Together with optical, fluorescence, and electron microscopy, high-speed-AFM provided evidence that the antibiotic causes the formation of toroidal pores or tubules in the membrane, killing the bacteria; or, alternatively, pore formation, which allows the membrane to release the pressure caused by the antibiotic’s action, maintaining the bacteria alive. In summary, by revealing the mechanisms by which Gram-positive and Gram-negative bacteria are altered by drugs and are able to resist them, AFM imaging provides a benchmark to fight against antimicrobial resistance.

## References

[1] Dufrêne YF (2017). Imaging modes of atomic force microscopy for application in molecular and cell biology. Nat. Nanotechnol..

[2] Viljoen A (2021). Force spectroscopy of single cells using atomic force microscopy. Nat. Rev. Methods Prim..

[3] Xiao J, Dufrêne YF (2016). Optical and force nanoscopy in microbiology. Nat. Microbiol..

[4] Dufrêne YF, Boonaert CJP, Gerin PA, Asther M, Rouxhet PG (1999). Direct probing of the surface ultrastructure and molecular interactions of dormant and germinating spores of phanerochaete chrysosporium. J. Bacteriol..

[5] Yamashita H (2012). Single-molecule imaging on living bacterial cell surface by high-speed AFM. J. Mol. Biol..

[6] Schabert FA, Henn C, Engel A (1995). Native Escherichia coli OmpF porin surfaces probed by atomic force microscopy. Science.

[7] Casuso I, Kodera N, Le Grimellec C, Ando T, Scheuring S (2009). Contact-mode high-resolution high-speed atomic force microscopy movies of the purple membrane. Biophys. J..

[8] Shibata M, Yamashita H, Uchihashi T, Kandori H, Ando T (2010). High-speed atomic force microscopy shows dynamic molecular processes in photoactivated bacteriorhodopsin. Nat. Nanotechnol..

[9] Yamashita H (2013). Role of trimer-trimer interaction of bacteriorhodopsin studied by optical spectroscopy and high-speed atomic force microscopy. J. Struct. Biol..

[10] Casuso I (2012). Characterization of the motion of membrane proteins using high-speed atomic force microscopy. Nat. Nanotechnol..

[11] Mulvihill E, Pfreundschuh M, Thoma J, Ritzmann N, Müller DJ (2019). High-resolution imaging of maltoporin LamB while quantifying the free-energy landscape and asymmetry of sugar binding. Nano Lett..

[12] Eskandarian HA (2017). Division site selection linked to inherited cell surface wave troughs in mycobacteria. Nat. Microbiol..

[13] Fantner GE, Barbero RJ, Gray DS, Belcher AM (2010). Kinetics of antimicrobial peptide activity measured on individual bacterial cells using high-speed atomic force microscopy. Nat. Nanotechnol..

[14] Alsteens D (2008). Organization of the mycobacterial cell wall: A nanoscale view. Pflüg. Arch. - Eur. J. Physiol..

[15] Viljoen A, Viela F, Kremer L, Dufrêne YF (2020). Fast chemical force microscopy demonstrates that glycopeptidolipids define nanodomains of varying hydrophobicity on mycobacteria. Nanoscale Horiz..

[16] Montero MT, Pijoan M, Merino-Montero S, Vinuesa T, Hernández-Borrell J (2006). Interfacial membrane effects of fluoroquinolones as revealed by a combination of fluorescence binding experiments and atomic force microscopy observations. Langmuir.

[17] Parsons ES (2019). Single-molecule kinetics of pore assembly by the membrane attack complex. Nat. Commun..

[18] Oh YJ, Plochberger B, Rechberger M, Hinterdorfer P (2017). Characterizing the effect of polymyxin B antibiotics to lipopolysaccharide on Escherichia coli surface using atomic force microscopy. J. Mol. Recognit..

[19] Manioglu, S. et al. Antibiotic polymyxin arranges lipopolysaccharide into crystalline structures to solidify the bacterial membrane. *Nat. Commun*. in press (2022). 10.1038/s41467-022-33838-0 PMC9587031 36271003

[20] Zuttion F (2020). High-speed atomic force microscopy highlights new molecular mechanism of daptomycin action. Nat. Commun..

